# Evaluation of the Olympus AU-510 analyser

**DOI:** 10.1155/S1463924691000366

**Published:** 1991

**Authors:** Carmen Farré, Jesús Velasco, Francisco Ramón

**Affiliations:** Servei de Bioquimica Hospital Sant Joan de Deu Carretera d'Esplugas Barcelona s/n 08034 Spain

## Abstract

The selective multitest Olympus AU-510 analyser was evaluated according to the recommendations of the Comision de Instrumentacion de la Sociedad Española de Quimica Clinica and the European Committee for Clinical Laboratory Standards. The evaluation was carried out in two stages: an examination of the analytical units and then an evaluation in routine work conditions. The operational characteristics of the system were also studied.

The first stage included a photometric study: dependent on the absorbance, the inaccuracy varies between +0.5% to -0.6% at 405 nm and from -5.6% to 10.6% at 340 nm; the imprecision ranges between -0.22% and 0.56% at 405 nm and between 0.09% and 2.74% at 340 nm. Linearity was acceptable, apart from a very low absorbance for NADH at 340 nm; and the imprecision of the serum sample pipetter was satisfactory.

Twelve serum analytes were studied under routine conditions: glucose, urea urate, cholesterol, triglycerides, total bilirubin, creatinine, phosphate, iron, aspartate aminotransferase, alanine aminotransferase and gamma-glutamyl transferase.

The within-run imprecision (CV%) ranged from 0.67% for phosphate to 2.89% for iron and the between-run imprecision from 0.97% for total bilirubin to 7.06% for iron. There was no carryover in a study of the serum sample pipetter. Carry-over studies with the reagent and sample pipetters shows some cross contamination in the iron assay.


Journal of Automatic Chemistry, Vol. 13, No. 5 (September–October 1991), pp. 217–220

### Evaluation of the Olympus AU-510 analyser

**Carmen Farré, Jesús Velasco and Francisco Ramón**
*Servei de Bioquimica, Hospital Sant Joan de Deu, Carretera d'Esplugas, s/n 08034 Barcelona, Spain*

*The selective multitest Olympus AU-510 analyser was evaluated according to the recommendations of the Comision de Instrumentacion de la Sociedad Española de Quimica Clinica and the European Committee for Clinical Laboratory Standards. The evaluation was carried out in two stages: an examination of the analytical units and then an evaluation in routine work conditions. The operational characteristics of the system were also studied.*

*The first stage included a photometric study: dependent on the absorbance, the inaccuracy varies between +0·5% to −0·6% at 405 nm and from −5·6% to 10·6% at 340 nm; the imprecision ranges between −0·22% and 0·56% at 405 nm and between 0·09% and 2·74% at 340 nm. Linearity was acceptable, apart from a very low absorbance for NADH at 340 nm; and the imprecision of the serum sample pipetter was satisfactory.*

*Twelve serum analytes were studied under routine conditions: glucose, urea urate, cholesterol, triglycerides, total bilirubin, creatinine, phosphate, iron, aspartate aminotransferase, alanine aminotransferase and gamma-glutamyl transferase.*

*The within-run imprecision (CV%) ranged from 0·67% for phosphate to 2·89% for iron and the between-run imprecision from 0·97% for total bilirubin to 7·06% for iron. There was no carry-over in a study of the serum sample pipetter. Carry-over studies with the reagent and sample pipetters shows some cross contamination in the iron assay.*

### Introduction

The Olympus AU-510 is a selective multichannel analyser for both routine and urgent analyses in medium to large laboratories. The present evaluation was carried out in accordance with the protocol of the Comision de Instrumentacion de las Sociedad Española de Quimica Clinica (SEQC) [1] and the guidelines of the European Committee for Clinical Laboratory Standards (ECCLS) [2].

The evaluation of the analytical units included a study of inaccuracy, within- and between-run imprecision, and photometric linearity from 340 to 410 nm, as well as the imprecision of serum sample pipetter.

The within- and between-run imprecision was studied for 12 serum analytes under routine conditions; carry-over in the serum and reagent sample pipetters was also examined.

In a practicability study, the versatility of the analyser, flexibility of the software, treatment of samples, technical training and preventative maintenance were evaluated.

### Materials and methods

### Instrument

The Olympus AU-510 (manufactured by Olympus Optical and distributed by Merck-Igoda S.A.) is a discrete, selective and multitest analyser. The analyser and the data processor are two separate units, with a hard disc of 20 Mb in the latter. The Olympus can hold up to 99 different tests and can be programmed to make 33 simultaneous tests on each sample. It can store results for up to 4000 patients, with a maximum of 60 analytes for each patient.

The results obtained cannot be compared with other instruments because the methodologies used vary and because the system is an open one.

The samples, held in a chain, can be introduced to the instrument in a primary tube or in a variety of secondary tubes; 100 samples can be run and more samples may be added at any time. The identification of the serum may be sequential or by bar code. The photometric unit has a diffraction-grating photometer and 10 differential filters.

In a rotor with 90 positions for cuvettes, the photometric measurements are made every 9 s. The cuvettes are used once only and the volume of the sample varies between 3 and 50 μl the reagent volume varies between 20 and 300 μl. The total volume of each cuvette is between 265 and 1150 μl.

The analyser contains a freezer where the reagents are held between 4°C and 10°C in 18 ml aliquots. Every test has up to nine positions for reagents which may or not be concentrated; three sets of 40 positions may be defined for reagent I (R–I) and three sets of 16 positions for reagent II (R–II).

### Reagents

Non-standard abbreviations are used here: M, Merck; S, Sigma; AST, aspartate aminotransferase (E.C. 2.6.1.1); ALT, alanine aminotransferase (E.C. 2.6.1.2); GGTP, γ-glutamyl transferase (E.C. 2.3.2.2); PNP, p-nitrophenol; NADH, β-nicotinamide adenine dinucleotide reduced form; NaOH, sodium hydroxide.

The following solutions were used to test the analytical units: PNP (Hopkin & Williams, 623800, Eurocontrol); NaOH (M,6498); from a solution of 360 μmol/l of PNP in 20 mmol/l of NaOH, different concentrations were obtained by dilution; disodium salt of NADH (S,N8129); Tris(hidroxymethyl)methylamine (M,8387); from a solution of 300 μmol/l of NADH in 80 mmol/l of Tris, different concentrations were obtained by dilution.

For tests on samples of sera the following reagent tests packs were used: glucose (M,14365M) (GOD-PAP); urea (M,19702) (urase/GIDH); urate (M,19739) (uricase-

0142–0453/91 $3.00 © 1991 Taylor & Francis Ltd.

*Table 1. Photometric inaccuracy.*

| | Theoretical absorbance | Mean observed absorbance* | Inaccuracy (%) |
|---|---|---|---|
| NADH 340 nm | 0·342 | 0·321 | −6·73 |
| | 0·685 | 0·648 | −5·64 |
| | 1·027 | 0·941 | −9·21 |
| | 1·370 | 1·270 | −7·88 |
| | 1·712 | 1·547 | −10·67 |
| PNP 410 nm | 0·610 | 0·613 | +0·51 |
| | 1·220 | 1·218 | −0·19 |
| | 1·830 | 1·793 | −2·10 |
| | 2·440 | 2·302 | −6·00 |

* Triplicate measurements.

PAP); cholesterol (M,19738) (CHOD-PAP); triglycerides (M,19706) (GPO-PAP); total bilirubin (M,19717) (DPD); creatinine (M,19726) (Jaffé, without deproteinization); phosphate (M,19723) (phosphomolybdate reduction without deproteinization); iron (M,19725) (ferrozine); AST (M,19708) (IFCC modified); ALT (M,19709) (IFCC modified); GGTP (M,19710) (γ-glutamyl-3-carboxy-4-nitroanilide).

For the calibration the following were used: Calibrator (M,19720) and for control sera: Qualitrol L (M,11307); Qualitrol N (M,11305) and Qualitrol H (M,11306).

### *Parameters evaluated*

#### *Photometric inaccuracy*

This is important when using a multiplying factor obtained from the molar absorptivity coefficient (e). Beginning with a solution of PNP (144 μmol/l) in NaOH (20 mmol/l) for the study at 410 nm, and a solution of disodium NADH (300 μmol/l) in Tris buffer (80 mmol/l) for the study of 340 nm, suitable dilutions and triplicate readings were made. Theoretical absorbance according to the molar absorptivity coefficient and the absorbance obtained were compared. The results were expressed as percentage according to the formula $[(V_t - V_0)/V_0] \times 100$. The Varian Cary-210 was used as a spectrophotometer of known inaccuracy (see table 1).

#### *Photometric imprecision*

The same solutions were used as in the previous section and the necessary dilutions were made to obtain approximate absorbances of 0·050, 0·100, 0·200, 0·500, 1·000, and 2·000; 30 readings in the same run were made of each dilution. The mean, standard deviation and coefficient of variation for each of the wavelengths (340 and 410 nm) were calculated (see table 2).

#### *Photometric linearity*

Triplicate measurements were made from an initial solution of 2·000 units of absorbance down to 0·000 with NADH at 340 nm and PNP at 410 nm. The percentage difference between the theoretical value and the value obtained for each of the dilutions was obtained. The dilutions were produced manually off-line and the analyses were realized in the same run (see tables 3 and 4).

*Table 2. Photometric imprecision.*

| | Mean absorbance | CV (%) |
|---|---|---|
| NADH 340 nm | 1·641 | 0·11 |
| | 0·888 | 0·10 |
| | 0·472 | 0·09 |
| | 0·200 | 0·11 |
| | 0·114 | 0·36 |
| | 0·057 | 2·74 |
| PNP 410 nm | 1·845 | 0·26 |
| | 0·943 | 0·36 |
| | 0·510 | 0·31 |
| | 0·206 | 0·22 |
| | 0·117 | 0·56 |
| | 0·061 | 0·45 |

* $N = 30$, within run.

*Table 3. Photometric linearity for NADH (340 nm).*

| Theoretical absorbance | Mean observed absorbance* | Dispersion (%) |
|---|---|---|
| 1·712 | 1·515 | −11·5 |
| 1·541 | 1·439 | −6·6 |
| 1·370 | 1·260 | −8·0 |
| 1·199 | 1·125 | −6·1 |
| 1·024 | 0·950 | −7·5 |
| 0·856 | 0·819 | −4·3 |
| 0·685 | 0·642 | −6·3 |
| 0·514 | 0·497 | −3·1 |
| 0·342 | 0·334 | −2·4 |
| 0·171 | 0·176 | +2·9 |
| 0·085 | 0·096 | +12·1 |
| 0·040 | 0·051 | +28·6 |

* Triplicate measurements.

*Table 4. Photometric linearity for PNP (410 nm).*

| Theoretical absorbance | Mean observed absorbance* | Dispersion (%) |
|---|---|---|
| 2·026 | 1·930 | −4·71 |
| 1·823 | 1·792 | −1·72 |
| 1·621 | 1·561 | −3·68 |
| 1·418 | 1·405 | −0·90 |
| 1·216 | 1·182 | −2·76 |
| 1·013 | 1·023 | +1·04 |
| 0·810 | 0·801 | −1·18 |
| 0·608 | 0·618 | +1·76 |
| 0·405 | 0·403 | −0·52 |
| 0·203 | 0·201 | −0·99 |
| 0·101 | 0·107 | +5·72 |
| 0·051 | 0·055 | +9·09 |

* Triplicate measurements.

*Table 5. Imprecision of the sample pipette delivery system (N = 30).*

| Sample pipette (µl PNP) | Reagent pipette (µl NaOH) | CV (%) |
|---|---|---|
| 3 | 250 | 1·78 |
| 18 | 250 | 0·75 |
| 27 | 250 | 0·98 |
| 33 | 250 | 0·89 |
| 48 | 250 | 1·14 |

*Sample pipette delivery system imprecision*

The following volumes were studied, based on the maximum and minimum volumes recommended by the manufacturer: min, (2 min + max)/3, min + max/2, (min + 2 max)/3, max. The sample was PNP at 3, 18, 27, 33 and 48 µl with a constant amount of 250 µl of NaOH (20 mmol/l). The PNP concentration was calculated in each case so that the final absorbance was approximately 0·500.

For the evaluation of performance under routine work conditions, the following parameters were studied (see table 5).

*Imprecision*

Within the same run, 30 samples of control sera were analysed at three levels, in order to obtain the within-run imprecision. The same control sera were analysed for 30 days, once per day, at three levels and were used to obtain the between-run imprecision.

*Sample-related carry-over*

In order to investigate the possible contamination between two consecutive sera through the serum sample pipetter, three high concentration sera were analysed: H1, H2 and H3, followed by three sera of low concentrations (L1, L2 and L3), 10 times for each analyte. The average value of (L1–L3) was calculated and its difference, h = L1–L3, needed to be less than 2SD of the within-run imprecision of that analyte.

*Specimen-independent carry-over*

To investigate the contamination through the reagent-reagent pipetter, it was necessary to study all the possible sequences of pipetting for the different reagents. The model used was: If A is the reagent studied and the rest are B, C, D, . . ., the following sequence should be constructed: A,A,A,B,A*,C,A*,D,A* etc. The carry-over effect has to be less than 2SD of the within-run imprecision per A*.

The results of this evaluation were:

*Imprecision*

The within- and between-run imprecision is summarized in table 6 and is acceptable for all the analytes studied; the lowest values are found for total bilirubin, phosphate and GGTP, and the highest for iron and creatinine. These analytes display very small changes of absorbance near the concentration of the calibrator which means that they have a poor reproducibility compared to the rest of the analytes evaluated.

*Sample-related carry-over*

In table 7 no contamination shows via the sample arm in the analytes studied. The difference between the concentration of the supposedly contaminated sample L1 and the non-contaminated L3 is practically nil for all the analytes.

*Specimen-independent carry-over*

All the pipetting sequences of the reagents studied show that there was no contamination between the different reagents, except in the case of the iron, which, when placed in front of the total proteins reagents, displays contamination (>2SD of the within-run imprecision).

### Practicability

This instrument is open for programming for different analytical techniques. It can carry out enzyme activities, kinetic or end-point determinations or methods with start reagents. It has an option for seven different models of mathematical calculation and accepts up to seven calibrators per technique. The reaction can be continuously monitored.

Preferential or urgent samples may be processed at all times through the STAT rotor which has eight positions. The first result of a preferential sample is obtained between 10·4 and 15 min after sampling.

Noteworthy features of the computer software are: the repetition program which allows any analyte to be repeated at any time and an ability to change the sample volume; the diagnostic program which checks the mechanical, electronic or optical performance of the instrument; the data-processing program which allows the demographic study of populations, correlation studies between analytes with presentation via graphics or histograms, printing of different lists, etc.; a program of quality control which defines up to 12 serum controls and allows a maximum of six controls per analyte. The instrument also permits storage of up to 60 days of quality control results and works with both prefixed limits and calculated values with visualisation of the results with Levy-Jennings or Youden graphics.

The analyser connected on-line through an RS-232 interface.

For technical staff training, one week is enough to learn the systematic work routine. For a deeper knowledge of the system, and for learning how to program it, one month should be sufficient.

The possibility of working with primary tubes avoids manipulation of the sample. On the other hand, one has to guard against the possible evaporation of serum when working with very small volumes in small wells for paediatric samples, given that the samples are placed in the analyser without caps. The process of sampling by chain means that small dilutions may be obtained

*Table 6. Within- and between-run imprecision for concentrations and enzyme activities.*

| | | Within-run ($N$ = 30) | | | Between-run ($N$ = 30) | | |
|---|---|---|---|---|---|---|---|
| | | Mean | SD | CV (%) | Mean | SD | CV (%) |
| | H | 11·82 | 0·11 | 0·93 | 10·80 | 0·13 | 1·25 |
| Glucose | M | 4·79 | 0·05 | 1·04 | 4·81 | 0·08 | 1·65 |
| (mmol. l-1) | L | 2·33 | 0·04 | 1·71 | 2·23 | 0·04 | 1·77 |
| | H | 34·42 | 0·49 | 1·42 | 27·60 | 0·47 | 1·69 |
| Urea | M | 7·08 | 0·09 | 1·27 | 6·90 | 0·09 | 1·29 |
| (mmol. l-1) | L | 2·53 | 0·03 | 1·18 | 2·60 | 0·04 | 1·54 |
| | H | 625·9 | 4·6 | 0·74 | 606·2 | 9·1 | 1·50 |
| Urate | M | 377·9 | 4·9 | 1·30 | 360·6 | 8·3 | 2·30 |
| (μmol. l-1) | L | 200·3 | 2·5 | 1·24 | 198·9 | 3·7 | 1·86 |
| | H | 6·30 | 0·05 | 0·80 | 6·13 | 0·10 | 1·59 |
| Cholesterol | M | 2·55 | 0·02 | 1·04 | 2·34 | 0·06 | 2·37 |
| (mmol. l-1) | L | 3·09 | 0·06 | 1·94 | 3·08 | 0·05 | 1·69 |
| | H | 2·78 | 0·03 | 1·08 | 2·69 | 0·06 | 2·12 |
| Triglyceride | M | 1·11 | 0·01 | 0·90 | 1·12 | 0·04 | 3·17 |
| (mmol. l-1) | L | 0·96 | 0·02 | 2·08 | 0·98 | 0·02 | 2·05 |
| | H | 70·57 | 0·53 | 0·75 | 68·66 | 0·66 | 0·97 |
| T. Bilirubin | M | 33·45 | 0·27 | 0·81 | 32·13 | 0·51 | 1·58 |
| (μmol. l-1) | L | 23·60 | 0·17 | 0·72 | 22·90 | 0·38 | 1·66 |
| | H | 565·5 | 4·8 | 0·84 | 637·0 | 16·02 | 2·51 |
| Creatinine | M | 184·5 | 2·6 | 1·41 | 187·9 | 4·84 | 2·57 |
| (μmol. l-1) | L | 113·5 | 2·9 | 2·55 | 113·4 | 3·25 | 2·86 |
| | H | 2·66 | 0·02 | 0·75 | 2·70 | 0·05 | 1·86 |
| Phosphate | M | 1·35 | 0·01 | 0·74 | 1·37 | 0·03 | 2·24 |
| (mmol. l-1) | L | 1·33 | 0·01 | 0·75 | 1·32 | 0·03 | 2·02 |
| | H | 34·87 | 0·62 | 1·77 | 32·93 | 0·01 | 3·05 |
| Iron | M | 27·16 | 0·28 | 1·03 | 26·63 | 0·89 | 3·36 |
| (μmol. l-1) | L | 15·81 | 0·46 | 2·91 | 16·24 | 1·15 | 7·06 |
| | H | 206·8 | 0·62 | 1·77 | 204·8 | 3·9 | 1·92 |
| AST | M | 120·4 | 0·99 | 0·83 | 114·8 | 3·2 | 2·82 |
| (UI. l-1) | L | 44·3 | 0·80 | 1·81 | 44·8 | 1·3 | 2·95 |
| | H | 161·9 | 1·52 | 0·94 | 169·1 | 4·5 | 2·69 |
| ALT | M | 78·0 | 0·68 | 0·87 | 80·1 | 2·5 | 3·14 |
| (UI. l-1) | L | 33·3 | 0·93 | 2·97 | 35·3 | 1·4 | 4·02 |
| | H | 131·0 | 1·03 | 0·78 | 133·1 | 2·6 | 1·95 |
| GGTP | M | 54·6 | 0·58 | 1·06 | 55·5 | 1·2 | 2·10 |
| (UI. l-1) | L | 24·1 | 0·31 | 1·28 | 24·5 | 0·8 | 3·20 |

*Table 7. Sample related carry-over.*

| *Concentration* | *High/low* | *h = L1–L3* |
|---|---|---|
| Glucose (mmol. l-1) | 10·9/2·33 | 0 |
| Urea (mmol. l-1) | 28·1/2·61 | 0·04 |
| Urate (mmol. l-1) | 606/215 | 0 |
| Cholest. (mmol. l-1) | 6·0/3·03 | 0·01 |
| Triglycer. (mmol. l-1) | 2·86/1·06 | 0 |
| Total Bil. (mmol. l-1) | 76·9/25·1 | 0 |
| Creatinine (μmol. l-1) | 628/109 | 0 |
| Phosphate (mmol. l-1) | 2·60/1·28 | 0 |
| Iron (μmol. l-1) | 32·4/15·1 | 0 |
| AST (Ul. l-1) | 225/44 | 1 |
| ALT (Ul. l-1) | 170/42 | 0 |
| GGPT (Ul. l-1) | 132/24 | 0 |

Contamination was not found if 'h' is less than 2 SD of the within run imprecision.

through the program of repetitions, while large dilutions have to be made manually.

The daily, weekly and monthly maintenance carried out by the user is minimal but it is important to the efficient functioning of the equipment. The quality of the water used has to be optimal, particularly when working with concentrated reagents.

### References

1. Comision de Instrumentacion del Comite Cientifico de al Sociedad Espanola de Quimica Clinica, Protocolo de evaluacion de Analizadores Automaticos: Evaluacion de los Modulos Analiticos, Doc.E, ver.3; Bol. Inf. SEQC (1986), No. 34, pp. 3–17.
2. European Committee for Clinical Laboratory Standards, Guidelines for the Evaluation of Analysers in Clinical Chemistry, ECCLS Document Vol. 3, No. 2 (June 1986).

